# Opto-electromechanical quantification of epithelial barrier function in injured and healthy airway tissues

**DOI:** 10.1063/5.0123127

**Published:** 2023-01-11

**Authors:** Jiawen Chen, Mohammad Mir, Maria R. Hudock, Meghan R. Pinezich, Panpan Chen, Matthew Bacchetta, Gordana Vunjak-Novakovic, Jinho Kim

**Affiliations:** 1 Department of Biomedical Engineering, Stevens Institute of Technology, Hoboken, New Jersey 07030, USA; 2 Department of Biomedical Engineering, Columbia University, New York, New York 10027, USA; 3 Department of Surgery, Columbia University, New York, New York 10027, USA; 4 Department of Cardiac Surgery, Vanderbilt University, Nashville, Tennessee 37235, USA; 5 Department of Biomedical Engineering, Vanderbilt University, Nashville, Tennessee 37235, USA

## Abstract

The airway epithelium lining the luminal surface of the respiratory tract creates a protective barrier that ensures maintenance of tissue homeostasis and prevention of respiratory diseases. The airway epithelium, unfortunately, is frequently injured by inhaled toxic materials, trauma, or medical procedures. Substantial or repeated airway epithelial injury can lead to dysregulated intrinsic repair pathways and aberrant tissue remodeling that can lead to dysfunctional airway epithelium. While disruption in the epithelial integrity is directly linked to degraded epithelial barrier function, the correlation between the structure and function of the airway epithelium remains elusive. In this study, we quantified the impact of acutely induced airway epithelium injury on disruption of the epithelial barrier functions. By monitoring alternation of the flow motions and tissue bioimpedance at local injury site, degradation of the epithelial functions, including mucociliary clearance and tight/adherens junction formation, were accurately determined with a high spatiotemporal resolution. Computational models that can simulate and predict the disruption of the mucociliary flow and airway tissue bioimpedance have been generated to assist interpretation of the experimental results. Collectively, findings of this study advance our knowledge of the structure–function relationships of the airway epithelium that can promote development of efficient and accurate diagnosis of airway tissue injury.

## I. INTRODUCTION

**FIG. 1.**
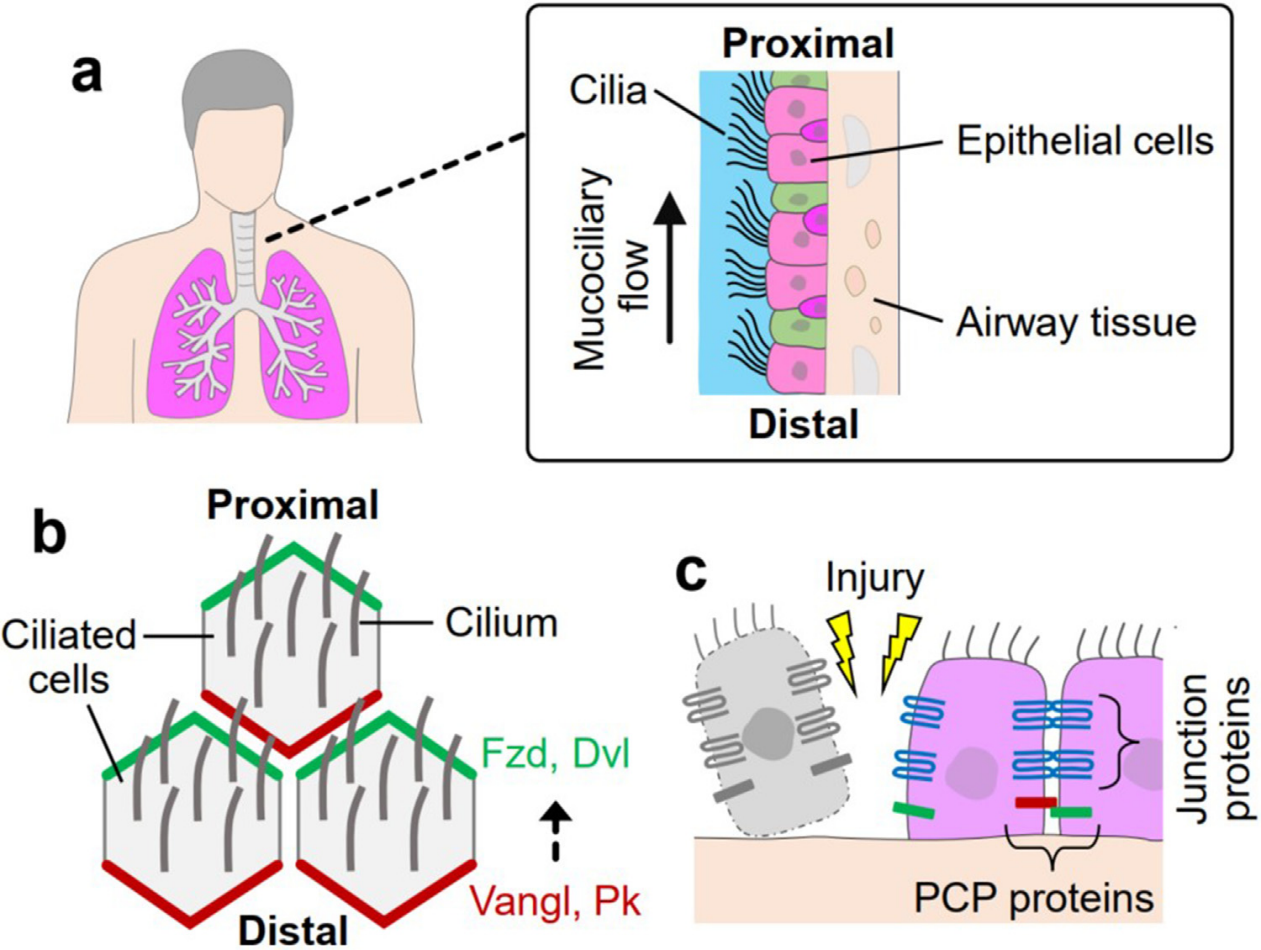
Airway epithelial barrier established by polarized, coordinated arrangement of airway epithelium. Schematics of (a) unidirectional mucociliary flow generated by cilia beating and (b) polarized arrangement of airway epithelium along the proximal and distal axis via planar cell polarity (PCP) signal pathway. Fzd: frizzled, Dvl: dishevelled, Vangl: Van Gogh-like, Pk: Prickle. (c) Schematic of epithelial barrier disruption caused by airway tissue injury.

The airway epithelial cells lining the luminal surface of the respiratory tract collectively generate protective barrier functions, including mucociliary (MCC) clearance,^1^ intercellular junction formation,^2^ and antimicrobial secretion.^3^ In particular, the mucociliary flow is a unidirectional surface flow that moves across the airway lumen to ensure clearance of the inhaled materials.^4^ This unidirectional flow is produced in the direction from the distal (D) to proximal (P) lung regions by coordinated, repeated, and polarized beating of cilia of multiciliated cells [Fig. 1(a)].^5,6^ Polarized arrangement of these cells along the P–D axis, which is known as planar cell polarity (PCP), ensures generation of the unidirectional flow.^6,7^ Tissue-level PCP is established and maintained by Frizzled (Fzd)/Dishevelled (Dvl) and Van Gogh-like (Vangl)/Prickle (Pk) protein complexes that are arranged at the proximal and distal cell membrane domains, respectively [Fig. 1(b)].^6–8^ Furthermore, tight and adherens junctions formed between epithelial cells play an important role in the generation of the defensive epithelial barrier.^9^ Transmembrane proteins, such as Zonula occludens-1 (ZO-1) and E-cadherin, promote tight connection between the membranes of adjacent epithelial cells, regulating paracellular permeability.^10^

As the first defensive mechanism of the lung, the epithelial barrier is frequently challenged and injured by inhaled harmful particles, gases, or pathogens,^11^ or through physical trauma that occurs during clinical procedures, such as intubation and anastomosis [Fig. 1(c)].^12^ While a minor injury can be resolved quickly by intrinsic repair mechanisms of the healthy airway tissue, severely or repeatedly injured epithelium (EP) can cause prolonged or permanent failure in repair or reduced level of restoration relative to normal tissue integrity and functions.^13^ Disruption in the tissue repair and regeneration pathways and loss of the epithelial cells' ability to restore the defensive barrier could result in aberrant remodeling and structural damage that can further impair epithelial functions.^14^ Acute or persistent structural damage induced to the airway tissue is known to degrade the epithelial barrier functions. However, the correlation between the structure and function of the airway epithelium remains largely elusive.

This study aimed to elucidate the impact of acute airway injury on epithelial barrier functions, including mucociliary clearance and tight/adherens junction formation. To this end, freshly harvested rat tracheal tissues have been studied *ex vivo* to assess the relationships between the epithelial structure and barrier functions of both healthy and acutely injured airway tissues. The *ex vivo* airway tissues retained the intrinsic epithelial barrier functions and structure, cellular composition, and underlying extracellular matrix (ECM) components, allowing accurate assessments of the airway tissues. To quantitatively evaluate the epithelial barrier dysfunction, we induced both localized and global injury with varying degrees to the isolated rat tracheas that recapitulates the structural and functional disruption in the injured airway epithelium. The impact of the injury on the mucociliary clearance function of the epithelium was assessed via real-time tracking of microparticles (MPs) being transported over the surface of the airway lumen. Furthermore, we confirmed that the epithelial injury can be diagnosed *in situ* by measuring electrical properties of the local airway tissue directly at the injury site. To rationalize the acquired experimental data and identified epithelial structure–function relationships, we created computational models that can numerically simulate and predict the impairment of the epithelial barrier functions.

Overall, this study has established both experimentally and computationally the correlations between the structural integrity and functional output of the airway epithelial barrier. The results of the study can contribute to the development of an innovative opto-electromechanically enabled diagnostic modalities coupled with computational models that can accurately quantify and predict initiation, progression, and resolution of the epithelial injury.

## II. RESULTS

### A. Generation of localized focal injury in the airway epithelium

**FIG. 2.**
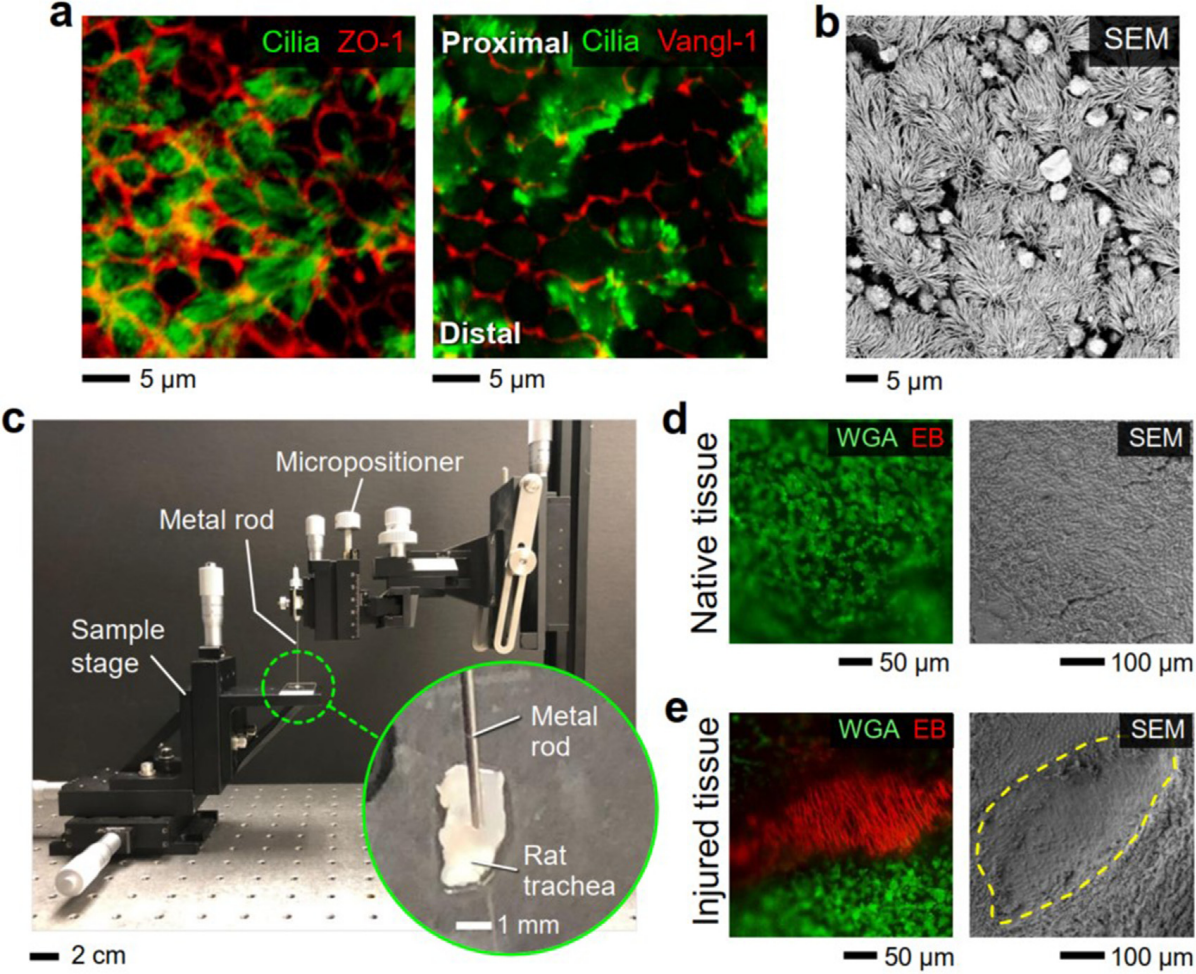
Induction of localized acute physical injury to rat trachea epithelium *ex vivo*. (a) Rat trachea whole-mount stained for tight junction protein Zonula occludens-1 (ZO-1), acetylated α tubulin to label cilia, and PCP protein Vang-like1 (Vangl-1). (b) Scanning electronic microscopy (SEM) image showing the luminal surface of the rat trachea. (c) Experimental setup used to generate the airway tissue injury. Localized epithelial injury was induced by gently compressing the tissue with the tip of a thin metal probe (diameter: 500 *μ*m). Fluorescence and SEM images of (d) native and (e) injured lumen of rat tracheal tissues. Ciliated cells were labeled with WGA (wheat germ agglutinin; green), while the injured tissue was visualized by EB (Evans blue; red). Injury site is indicated with an yellow dotted line.

To recapitulate localized mucociliary disruption caused by a focal epithelial injury, we generated an *in vitro* airway injury model (Fig. 2). Immunostaining analysis of healthy rat airway tissues revealed abundant cilia of multiciliated cells, intercellular ZO-1 tight junctions between the epithelial cells, and polarized localization of Vangl-1 PCP protein along the P–D axis [Fig. 2(a)]. Similarly, scanning electron microscopy (SEM) images of the healthy airway tissues showed densely populated multiciliated and secretory cells across the entire surface of the airway lumen [Fig. 2(b)]. We then generated a localized physical injury in the epithelium of isolated rat trachea tissue using a metal rod that was mounted on a micropositioner [Fig. 2(c)]. Real-time visualization of the epithelial integrity and cilia function was achieved by staining the cilia of freshly isolated airway tissues with fluorescein-labeled wheat germ agglutinin (FITC-WGA) prior to experiment. Furthermore, structural integrity of the airway tissue surface was monitored via SEM imaging and H&E staining following all experiments [Figs. 2(d) and 2(e); supplementary material Figs. 1 and 2)]. While native airway tissue showed abundant cilia that continuously beat along the P–D axis [Fig. 2(d); supplementary material Video 1), fibrous connective tissue that was stained with Evans blue (EB) was observed at the injured tissue region [Fig. 2(e)]. Post-analysis of the airway tissue with SEM imaging, immunostaining, and H&E staining showed localized damage on the epithelial layer and absence of tight/adherens junctions at the injured tissue region, while other tracheal layers were preserved [supplementary material Figs. 1(a), 1(b), and 2].

### B. Quantification of mucociliary flow disruption caused by acute epithelial injury

**FIG. 3.**
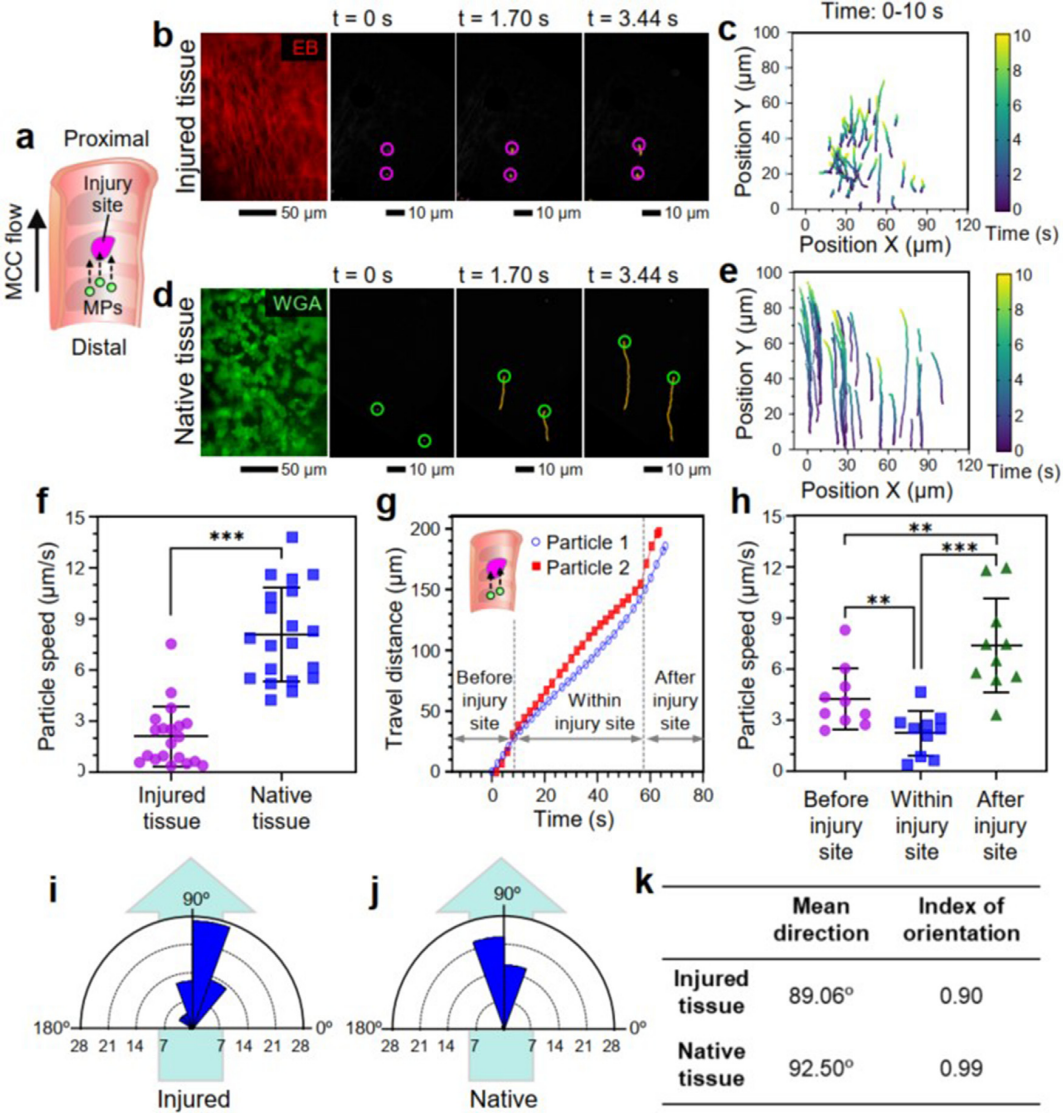
Quantification of the speed and direction of cilia-generated flow in native and injured airway via microparticle tracking. (a) Schematic of mucociliary (MCC) flow and microparticle movement along the proximal–distal axis of isolated rat trachea. MPs (microparticles). (b) and (c) Fluorescent images of injured tracheal luminal surface and trajectories of microparticles traveling on the injured epithelial surface. EB (Evans blue). (d) and (e) Fluorescent images of native tracheal luminal surface and trajectories of microparticles placed on the intact surface. WGA (wheat germ agglutinin). (f) Microparticle traveling speeds determined by monitoring the trajectories of the particles at injured and native tissue regions, respectively. ^***^p <0.001. (g) Travel distance and (h) average traveling speeds of microparticles measured as the particles moved across the injury site. Polar histograms showing the angular direction of the moving microparticles on (i) the injured and (j) native airway lumen. (k) Mean angular direction and index of orientation of the microparticles calculated through the particle trajectory analysis. ^**^p <0.01 and ^***^p <0.001.

We investigated the extent of the mucociliary flow affected by the localized airway epithelial injury (Fig. 3). To this end, the speed and direction of the mucociliary flow at both acutely injured and native tissue regions were monitored by tracking 1-*μ*m microparticles using a fluorescent microscope [Figs. 3(a)–3(e)]. The acquired videos were processed to determine the trajectory of each microparticle. The obtained trajectories revealed that the microparticles at the injured epithelial surface moved shorter distances [Figs. 3(b) and 3(c); supplementary material Video 2] than the microparticles at the ciliated tissue surface [Figs. 3(d) and 3(e); supplementary material Video 3] over a given amount of time (e.g., 10 s). The average speed of the microparticles measured at the injured region of damaged airway tissue (2.93 ± 1.15 *μ*m/s) was approximately 60% lower than those measured from the native tissue (7.13 ± 1.07 *μ*m/s) (^***^p <0.001) [Fig. 3(f)]. We were able to continuously trace some of the microparticles as they passed through the injury site, and the travel distance of the traced particles were plotted as a function of time [Fig. 3(g)]. The speed of particles, which is indicated by the slope of the curve, decreased as the particles approached the injury site and increased back to nearly normal range after passing the injury site. In general, the average speed of microparticles just before entering the injury site was approximately 4.26 ± 1.80 *μ*m/s, which was lower than the particle speed measured from the native tissue (7.13 ± 1.07 *μ*m/s). The speed of microparticles decreased to 2.23 ± 1.30 *μ*m/s within the injured region and increased to 7.29 ± 2.78 *μ*m/s, which was similar to the normal speed, just after passing the injury site [Fig. 3(h)].

We then studied how the traveling direction of the mucociliary flow was affected by the tissue injury by measuring the angle between each microparticle trajectory and the P–D axis of the airway [Figs. 3(i) and 3(j)]. Measured angles were plotted in a polar histogram, in which the angular direction of each pie-shaped wedge represents the microparticle moving direction (e.g., microparticles traveling toward the proximal direction along the P–D axis was set to 90°). The size of a wedge corresponds to the number of microparticles moving in that direction. Results showed that all microparticles tend to move toward the proximal direction in both injured and native tissues. In the injured tissue, only 22.41% of the traced microparticles moved between 85° and 95° of the angular direction [Fig. 3(i)], whereas 62.50% of the microparticles in the native tissue traveled between 85° and 95° [Fig. 3(j)]. The mean microparticle traveling direction was calculated to be 89.06° and 92.50°, respectively, in injured and native tissues. To further quantify the degree of unidirectionality based on the observations, we calculated the index of orientation of the traveling microparticles (see Sec. IV). The index of orientation for injured and native tissues were determined to be 0.90 and 0.99, respectively, suggesting approximately 10% reduction in the unidirectional movement of the mucociliary flow in injured airway tissue [Fig. 3(k)]. We occasionally observed a lateral movement of the surface fluid at the boundary parallel to the P–D axis between the injured and intact tissue regions (supplementary material Fig. 3; supplementary material Video 4).

### C. Computer simulation of the injury-induced mucociliary flow disruption

**FIG. 4.**
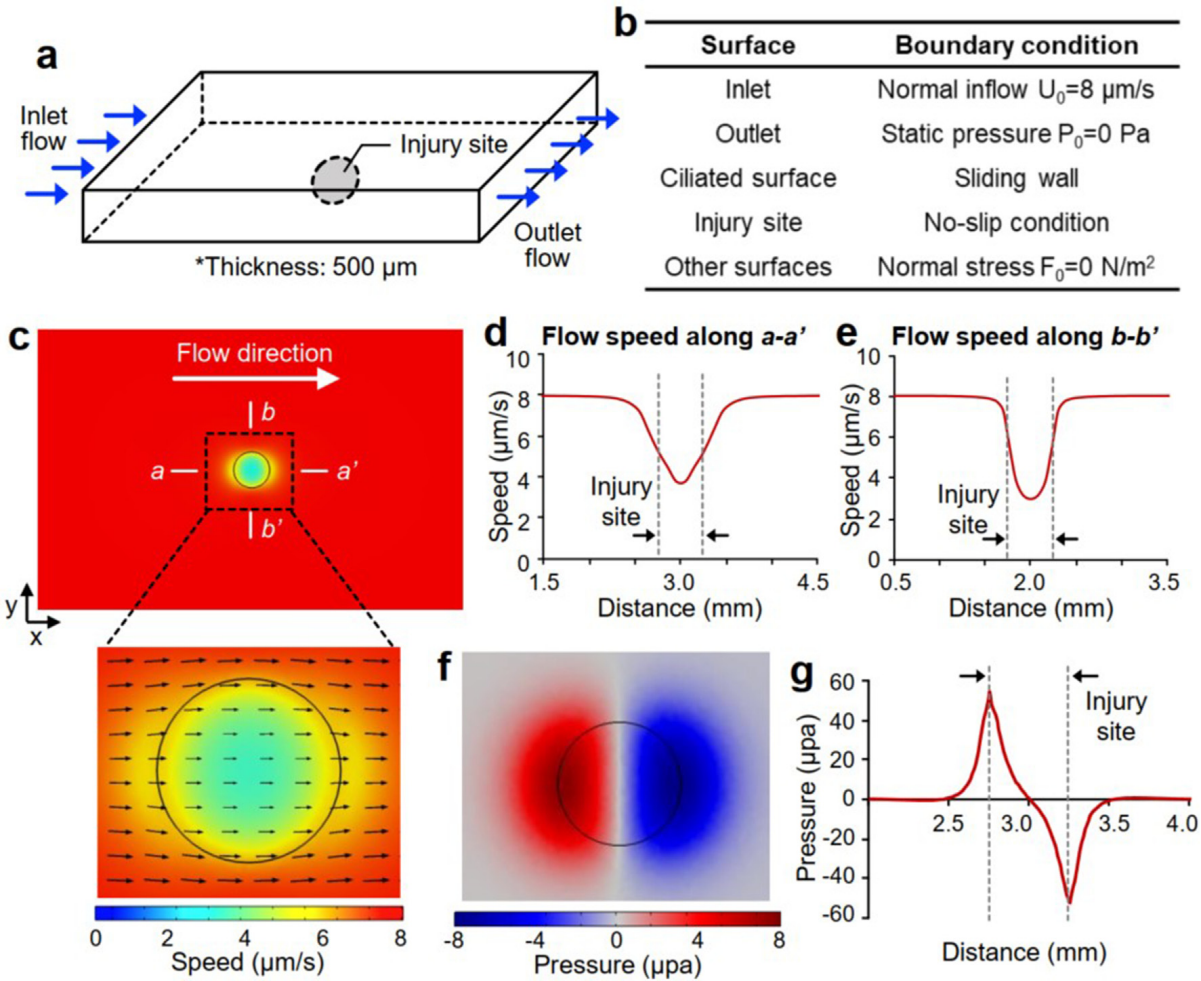
Computer simulation of mucociliary flow near the injury site. (a) Schematic of the three-dimensional (3D) geometry of the mucociliary flow simulated using COMSOL software. (b) Boundary conditions and values applied at different surfaces of the computational model. (c) Simulation results showing the flow speed and velocity vectors near a circular injury site. Computer simulated speed of the flow measured along (d) a–a′ and (e) b–b′ lines that are indicated in (c). (f) Pressure distribution within the flow at the injury site indicated with a black circle and (g) magnitude of the pressure inside the flow calculated along a–a′.

To rationalize the experimental results, we simulated the mucociliary flow near the injured airway luminal surface (Fig. 4). Mucociliary flow was modeled as a thin surface flow (thickness: 500 *μ*m) that passed over the injured airway lumen [Fig. 4(a)]. The flow entered the simulated region through the inlet surface at a normal speed of 8 *μ*m/s and exited through the outlet. The intact ciliated airway surface was modeled as a unidirectional sliding wall (speed: 8 *μ*m/s) to recapitulate the flow of the thin airway surface liquid whose movement is driven by the continuous polarized ciliary motion. No-slip boundary condition (BC) was applied to the injured tissue region where the coordinated ciliary activity is lacking [Fig. 4(b)].^15^ The simulated flow, which was initially traveling at 8.00 *μ*m/s, traveled slower in the region adjacent to the damaged area [Fig. 4(c)], and its speed further declined to 3.75 *μ*m/s at the central region of the injury site [Figs. 4(d) and 4(e)]. The flow speed returned to the normal level (8.00 *μ*m/s) after passing the injury site. The pattern of the flow speed change near the injury site is consistent with our experimentally observed results. In our experiments, the speed of particles at the normal tissue, injury site, and downstream region was 7.13 ± 1.07, 2.23 ± 1.30, and 7.29 ± 2.78 *μ*m/s, respectively (Fig. 3). Furthermore, as the flow approached the injury site, the hydrodynamic pressure within the fluid increased from 0 Pa to 53.5 *μ*Pa because of the reduced flow speed. As the flow passed the injury site, the pressure reduced back to 0 Pa at the center of the injury site. Then, the pressure further decreased to −53.5 *μ*Pa and returned to 0 Pa as the flow continued to travel across the injury site [Figs. 4(f) and 4(g)].

### D. Quantification of the effects of acute tissue injury on alteration of tissue bioimpedance

**FIG. 5.**
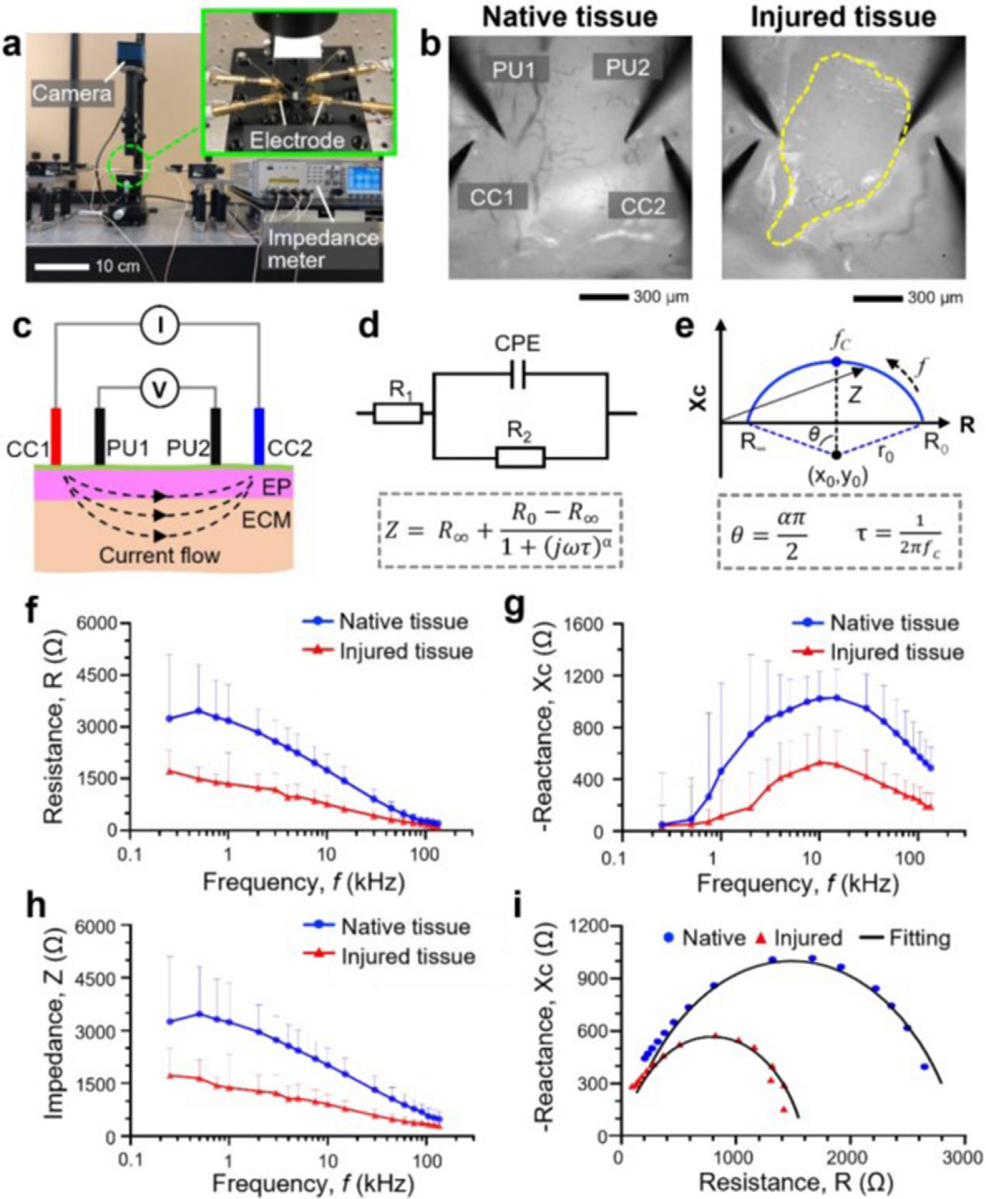
Bioimpedance measured in injured and intact rat trachea airway tissues. (a) The experimental setup used to measure bioimpedance of the airway tissue. (b) Bright field images show the luminal surfaces of native and injured trachea tissues. Dotted line indicates the injured tissue region. CC1 and CC2: current carrying electrode 1 and 2, PU1 and PU2: voltage pickup electrodes 1 and 2. (c) Schematic of the cross-sectional view of the rat trachea with four-probe bioimpedance measurement system. I: Current source, V: voltage meter, EP: epithelium, ECM: extracellular matrix. (d) An equivalent electrical circuit of the rat trachea, known as the Cole model. R_1_ and R_2_: resistor 1 and 2, CPE: constant phase element, Z: impedance, R_0_: resistance at zero frequency, R_∞_: resistance at infinite frequency, α: an empirical exponent (range: 0 and 1), τ: characteristic time constant, R_1_ = R_0_, R_2_ = R_0_–R_∞_. (e) Cole–Cole plot that graphically shows different parameters. f_c_: Frequency corresponding to the maximum reactance. (f) Resistance (R), (g) reactance (X_C_), and (h) impedance (Z) of both native and injured trachea tissues measured with different alternating current (AC) frequency values (range: 250 Hz–135 kHz). (i) Complex impedance plot of native and injured tracheas. The solid lines represent the best fit to the measured data for the extraction of Cole parameters.

We investigated whether the localized damage created in the airway epithelium can be detected via measurement and analysis of the electrical property at the injury site (Fig. 5). To this end, we created a measurement platform that allowed us to quantify the bioelectrical impedance (i.e., bioimpedance) of the airway tissue [Fig. 5(a)]. This platform utilizes the four-point measurement approach, in which four electrode probes directly contact the airway tissue to measure the electrical properties of the tissue. For the experiment, we created a focal epithelial injury using a metal rod, and the four electrodes were placed near the injury site [Fig. 5(b)]. During the measurements, alternating electrical current (AC) with various frequencies (range: 250 Hz–135 kHz) and 100 *μ*A of its maximum amplitude were injected into the tissue using two current carrying electrodes (CC1 and CC2), while two voltage pickup electrodes (PU1 and PU2) measured the potential distribution at the measurement site [Fig. 5(c)]. The data acquired were then analyzed using the Cole model that describes the biological tissue as a combination of two resistors (R_1_ = R_∞_ and R_2_ = R_0_–R_∞_) and a constant phase element (CPE)^16,17^ (see Sec. IV for details) [Figs. 5(d) and 5(e)].

We then tested the hypothesis that disruption of the airway epithelium leads to reduced bioimpedance of the airway tissue measured at the airway lumen. The measurement results showed that the magnitude of both resistance (R) and reactance (Xc) measured at the injured tissue was noticeably lower than that measured at the native tissue region [Figs. 5(f) and 5(g)]. The magnitude of the reactance increased with increased AC frequency (from 0 to ∼500 Ω in the injured tissue; from 0 to ∼1000 Ω in the native tissue) and then gradually decreased as the frequency further increased after a certain threshold frequency (*f*c: ∼10 kHz in the injured tissue; ∼15 kHz in the native tissue) [Fig. 5(g)]. The bioimpedance (Z) of both injured and native airway tissues reduced gradually as the AC frequency increased [Fig. 5(h)]. Each dataset acquired from the injured and native tissues was fitted to a curve by least absolute deviation (LAD) method, from which the Cole parameters were extracted^18,19^ (see Sec. IV) [Fig. 5(i); supplementary material Table 1]. Notably, the resistance values measured at DC (R_0_) and infinite AC (R_∞_) and the time constant (τ) acquired from the injured tissue were smaller than those acquired from the native tissue, which is likely due to damaged airway epithelial barrier at the injury site.

### E. Computer simulation of bioimpedance of injured airway tissue

**FIG. 6.**
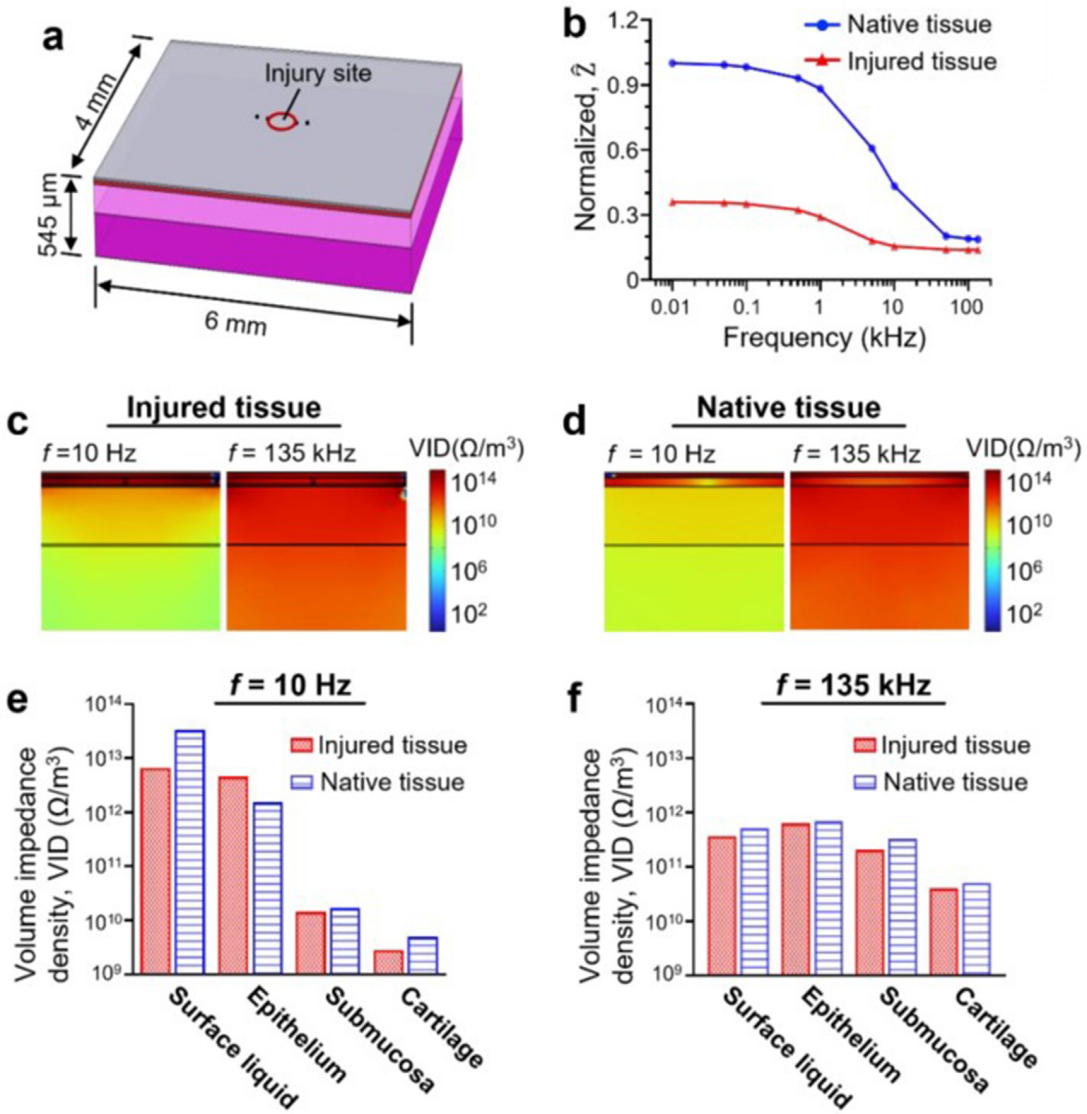
Computer simulation of bioimpedance of injured and native airway tissues. (a) 3D computer model of injured trachea used for the computer simulation. (b) Simulated values of normalized impedance ( $\hat{Z}$) of native and injured trachea with respect to AC frequency. Simulated volume impedance density (VID) profile of (c) injured and (d) native trachea tissue layer with AC frequency of 10 Hz and 135 kHz. VID values determined via the simulations at (e) 10 Hz and (f) 135 kHz.

To rationalize the contribution of the airway epithelial injury in the alteration of the electrical property of the airway tissue, we numerically simulated the electrical current propagation through both injured and native airway tissues (Fig. 6). The airway tissues were modeled as a composite of tissue layers that consisted of epithelium, submucosa, and cartilage. Furthermore, the computer model included a thin layer of pulmonary surface liquid (thickness: 15 *μ*m) deposited on top of the epithelial layer [Fig. 6(a); supplementary material Fig. 4].^20^ The computational simulation results showed a similar trend observed in the experiments where the potential difference (ΔV) (supplementary material Fig. 5) and normalized bioimpedance ( $\hat{Z}$) [Fig. 6(b)] between the voltage pickup electrodes (PU1 and PU2) decreased with increased AC frequency. Notably, the impedance values measured at the injured tissue were generally smaller than those measured from intact regions, which were consistent with our experimental results (Fig. 5). Contour plots of the simulated results showed that the distribution of the electric field (E) (supplementary material Fig. 6) and current density (J) (supplementary material Fig. 7) throughout the tissues were substantially affected by the AC frequency supplied to the tissue. While the electrical current propagates through the superficial tissue near the epithelium at lower AC frequency, the current can penetrate deeper tissue regions as it travels through the tissue at higher AC frequency.

**FIG. 7.**
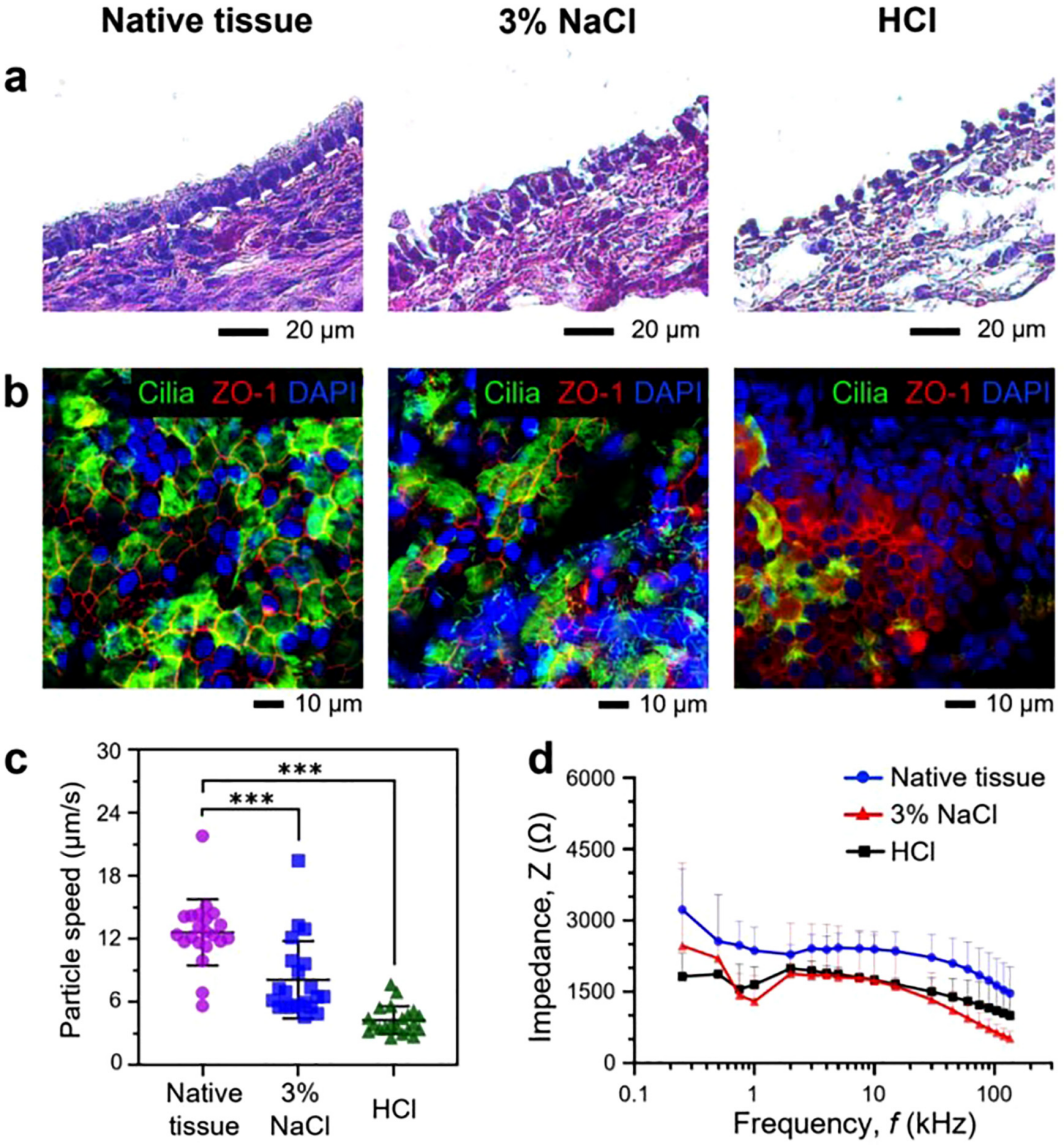
Opto-electromechanical quantification of inhalation-induced airway epithelium injury. (a) Microscopic analysis of native and injured trachea tissues via H&E staining. Injury was induced by inhalation of either 3% NaCl or HCl with pH 1.5. (b) Immunofluorescence staining of the tissues with ZO-1 (red), acetylated α tubulin to label cilia (green) and nuclei (blue). (c) Traveling speeds of traced microparticles measured from native and injured tissues. (d) Impedance (Z) measured from native and injured trachea tissues with the AC frequency range between 250 Hz and 135 kHz. ^***^p < 0.001.

To investigate relative contribution of each tissue layer in the electrical measurement outcomes, we computed volume impedance density (VID) of different tissue layers in both injured and native airway tissues [Figs. 6(c)–6(f)]. Since VID is defined as the magnitude of the bioimpedance per unit tissue volume, it can provide information about the relative role of different tissue layers in resistance to the electrical current. At low AC frequency (e.g., 10 Hz), the VID value in the surface liquid was three to four orders of magnitude greater than that in the submucosa or cartilage layer in both injured and native tissues [Fig. 6(e)]. VID value of the surface liquid in the injured tissue (6.58 × 10^12^ Ω/m^3^) at 10 Hz was much smaller than that in the native tissue (3.36 × 10^13^ Ω/m^3^) due to easier penetration of electrical current through the damaged epithelium than intact epithelium. The difference of VID between the superficial and distal tissue regions became less significant at higher AC frequency (e.g., 135 kHz), suggesting low AC frequency could generate more robust and reliable electrical readouts when characterizing the tissue injury that occurs at the superficial airway tissue layers [Fig. 6(f)].

### F. Opto-electromechanical quantification of airway barrier function disruption caused by inhalation-induced acute tissue injury

Following the thorough experimental and computational validations of our opto-electromechanical based methods using the airway tissue with focal injury, we then investigated whether less severe and more clinically relevant airway tissue injury can be assessed using this approach. To this end, we created an inhalation-induced airway injury model by intratracheally instilling either hypertonic saline or acidic solution (Fig. 7). Three percent sodium chloride (3% NaCl) solution was used as a hypertonic solution because its high osmotic pressure can disrupt the tight junctions of the airway epithelium, generating mild tissue injury with increased epithelium permeability.^21^ Furthermore, hydrogen chloride (HCl) with low pH level (pH 1.5) was used to generate moderate injury in the epithelium that mimics gastric acid aspiration injury.^22^

H&E and immunofluorescence staining were performed to confirm the establishment of epithelium injury with varying degrees [Figs. 7(a) and 7(b)]. H&E staining images showed that the native tissue contained intact epithelial layer, while the airway tissues exposed to 3% NaCl or HCl appeared to be considerably damaged. Notably, the severity of the injury in HCl-treated tissues was greater than NaCl-treated tissues as the H&E staining showed many of the epithelial cells that appeared to be dissociated from the tissue surface [Fig. 7(a)]. Similarly, immunostaining images clearly showed the establishment of inhalation-induced epithelium injury. Disruption of the ciliated cells (green) and tight junctions (red) were confirmed by decreased intensity or diffused pattern of associated fluorescence signals [Fig. 7(b)]. To more accurately quantify the tissue injury using the immunostaining images, we calculated the surface area of airway lumen covered with ciliated cells (green) in the native and injured tissues using ImageJ. In the native tissue, 44.43% of the luminal surface was covered with ciliated cells, while less than 22.91% and 13.03% of the airway lumen contained ciliated cells in the tissue treated with NaCl and HCl solutions, respectively (supplementary material Fig. 8).

With the inhalation-induced injury model established, we investigated whether the mild and moderate epithelium injury can be probed via the particle tracing and impedance recording methods [Figs. 7(c) and 7(d)]. Results showed that the average speeds of the microparticles measured from 3% NaCl-treated tissues (8.13 *μ*m/s) and HCl-treated tissues (4.29 *μ*m/s) were approximately 36% and 66% lower than those determined from the native airway tissue (12.72 *μ*m/s) (^***^p < 0.001), respectively [Fig. 7(c)]. Similarly, the magnitudes of the electrical impedance measured from the injured airway tissues were generally lower than those of the native tissues, most likely due to the considerable disruptions of the tight junctions in the injured tissues [Fig. 7(d)]. These results confirmed that our opto-electromechanical based method can provide quantitative information about airway epithelium with mild and moderate injury.

## III. DISCUSSION

In this study, we investigated how acute physical injury generated in the airway epithelium affects the intrinsic protective functions of the epithelial barrier and whether the functional disruption can be measured directly at the injured tissue regions. Specifically, we quantified the impact of injury-induced degradation of the mucociliary clearance and tight/adherens junction formation, which are the two most essential barrier functions of the airway epithelium. To quantitatively investigate the changes of these epithelial functions following acute injury, we used *in situ* particle tracking and bioimpedance measurement techniques (Figs. 3, 5, and 7), combined with mathematical modeling and computer simulation (Figs. 4 and 6). To enhance reliability of the study outcomes, we utilized freshly isolated rat tracheal tissues that retained the intrinsic airway tissue architecture and functions, including unidirectional ciliary activities, physiologic cell diversity and distribution, and the native tissue matrix components. Localized acute physical injury was created in a selected region of the isolated tracheal tissues to determine the correlation between structure and functions of the airway epithelium with improved spatiotemporal resolution of the measurements. Similarly, inhalation-induced epithelium injury was generated to evaluate the utility of this study in broader clinical conditions. The numerical analyses conducted in this study facilitated elucidation of the structure–function correlation of the airway epithelium by allowing interpretation of the experimentally obtained results.

Airway epithelial injury and subsequent impairment of the epithelial barrier functions can be caused by various reasons, such as inhalation injury, trauma, and surgery. The extent of the tissue damage and functional impairment can drastically vary depending on the cause, type, and severity of the injury. Accordingly, we focused on analyzing the tissue structure and function following different degrees of damages induced to the isolated airway mucosal tissues by either physical or chemical treatment. This injury model recapitulates the structural and functional disruption in the epithelium that nearly all pathologies of airway injury have in common with varying degrees.^12,23^

The tissue-wise particle tracking revealed that the localized tissue injury substantially degraded the ability of the airway mucosa to transport its surface fluid as the speed of the flow reduced by nearly 60% at the injured tissue regions. Furthermore, the injury resulted in disorientation of the surface flow as the unidirectionality of the flow reduced by at least 10% near the central location of the injury site (Fig. 3). Computer simulation of the injury-induced fluid movements showed that the spatial variation of the flow speed near the injury site is likely due to the heterogeneous pressure distribution resulting from the absence of the functioning cilia at the injured tissue (Fig. 4). These findings are consistent with a pathological condition where airway tissue regions with substantially impaired mucociliary clearance are venerable to subsequent injury and infection due to increased time for the inhaled particles or pathogens to be removed from the respiratory tract.^24^

While the flow speed and directionality reduced, transport of the surface fluid could persist at the tissue regions devoid of ciliated cells. This is because the fluid moves as a continuous medium due to intermolecular attractive forces between the fluid molecules.^25^ Because of the physical connection between the fluid molecules across the airway surface, the hydrodynamic energy produced by repeated cilia beating at the neighboring intact tissue region can propagate through the fluid due to energy conservation, leading to fluid movement at the injured region. The hydrodynamic crosstalk between the fluids at different locations was clearly visualized via particle tracking at the interface between the intact and injured tissue regions (supplementary material Fig. 3; supplementary material Video 4). Due to substantial differences between these two regions in their flow speed and directionality, the surface flow moving over the intact tissue surface caused movement of the fluid from the injury site toward the intact region in the direction perpendicular to the P–D axis of the airway. Propagation of hydrodynamic energy within a fluid is heavily influenced by the fluid properties, such as viscosity, surface tension, and density.^26^ Therefore, the maximum size of the injured tissue surface that neighboring cilia can exert their effects is expected to be dependent on the physical characteristics of the airway surface fluid.

Visualization of the mucociliary flow and cilia motion can be achieved via advanced imaging modalities, in particular, optical coherence tomography (OCT).^27^ OCT imaging, however, can only provide the cross-sectional views of tissues or fluids and thus is not suitable for monitoring the two-dimensional (2D) mucociliary flow across large airway surface. On the contrary, the current study employed a microparticle tracking method, which has been extensively used in the mucociliary-related studies,^15,28^ because of its proven utility in visualization of fluid movements with high spatial and temporal resolution. Tracking the movement of individual microparticles simultaneously along the 2D surface of the injured airway lumen allowed us to rapidly assess the impact of localized tissue damage on the mucociliary flow. Furthermore, assessment of the fluid interactions between the injured and intact tissue regions, which can be difficult to visualize using OCT imaging, was realized via the *in situ* particle tracking approach implemented in this study.

We further showed that disruption of the tight/adherens junctions between the epithelial cells caused by the physical tissue damage can be quantified by measuring the electrical properties of the local airway tissue (Fig. 5). Notably, we determined that the acute tissue injury resulted in reduced bioimpedance using our custom-built measurement platform in combination of the mathematical analysis and computer simulation (Figs. 5 and 6). Loss of the physically protective and electrically resistant epithelial barrier is likely the main contributor of the lowered magnitude of the bioimpedance of the injured airway tissues compared with that of native tissues.^29,30^ Consistent with prior studies, our experimental and simulation results showed that the magnitude of the electrical resistance of both native and injured tissues decreased with increased AC frequency because the dielectric property of the cell membrane hampered propagation of AC with lower frequency across the cells.^31,32^ In particular, the simulation results showed that the electrical current can propagate into deeper tissue regions when the resistant epithelial layer was removed, suggesting the reduced bioimpedance measured at the injured airway surface is likely due to diffusive distribution of the electrical potential across the larger volume of the airway tissue. Since measuring the electrical characteristics of each tissue layer is very challenging because of their thin geometry (supplementary material Fig. 5), the computer model developed can mathematically predict the electrical response of individual tissue components, providing additional quantitative information about the structural integrity of the airway tissue.

Following the rigorous experimental and computational validations of our epithelium assessment modality using the focal injury model, we generated inhalation-induced mild and moderate injuries by intratracheally instilling 3% NaCl and HCl (pH 1.5), respectively. The injured tissues were analyzed via histology, immunostaining, particle tracing, and impedance recording. Results indicated that the severity of the tissue injury was correlated with the degree of the structural and functional disruption created in the injured airway mucosa (Fig. 7; supplementary material Fig. 8). Furthermore, the study confirmed that our opto-electromechanical based method can accurately detect and quantify minor tissue damage, demonstrating its potential utility in the diagnosis of airway tissue injury.

Different types of bioimpedance measurement techniques have been developed and used extensively to evaluate the structure, content, and function of biological tissues and organs.^17,30,31^ For example, electrical impedance tomography (EIT) is an imaging modality that can visualize internal organs, such as lung, by mapping electrical properties of the patient's body in a noninvasive and radiation-free manner.^33^ Similarly, the percentages of body fat and muscle mass can be estimated indirectly by measuring the bioimpedance across a human body.^16^ Despite the wide usage of bioimpedance measurement methodologies, to the best of our knowledge, our study is the first to correlate airway tissue injury with bioimpedance. Significantly, the measurement and simulation outcomes suggest that the *in situ* bioimpedance quantification, when coupled with conventional bronchoscopy, could offer insights into mechanisms of airway epithelial destruction, or serve as a diagnostic tool to detect airway injury and disease. For instance, in clinical applications, flexible electrode probes or thin film-based electrodes incorporated into a video bronchoscope can be introduced into the respiratory tract of a patient with airway injury. By measuring the electrical properties of the patient's local airway mucosa, severity of the tissue injury could be accurately quantified.

In this study, the cyclic air flow movement generated in the lung due to breathing has not been simulated. The unidirectional mucociliary flow is created collectively by ciliated and secretory cells lining the surface of the respiratory tract. A number of studies suggest that the synchronized unidirectional ciliary movement and the direction of the subsequently generated mucociliary flow are established during prenatal stages of lung development.^7^ Furthermore, in healthy adult lungs, the fully established beating direction and synchronization of the ciliated cells are not changed by air flow.^34^ In this study, we used tracheal tissues that are freshly isolated from healthy adult rats, and all experiments were completed within three hours while the airway tissue remained viable and functional. Therefore, although the *in vivo*-like air flow has not been incorporated in our current system, it is unlikely that inclusion of the air flow would have significant effects on the mucociliary flow and tissue impedance.

Furthermore, for the particle tracing experiments in this study, the airway luminal surface was coated with exogenous liquid carrying microparticles that could alter the native air–liquid interface (ALI) condition of the airway. We distributed fluorescent microparticles across the epithelial surface via Dulbecco's modified Eagle cell culture medium. Addition of the particle-load liquid can result in disruption of the native ALI established on the airway surface by changing the physical properties and features of the surface lining fluid, such as viscosity, surface tension, and thickness. Since the speed of cilia-driven flow is influenced by the fluid properties,^35,36^ it is expected that the speeds of the mucociliary flow measured in this study could be different from those measured within the lung of live animals. *In situ* imaging methods, such as OCT, that can allow label- and particle-free cilia visualization could be used to investigate the role of ALI in the mucociliary flow characteristics.

In conclusion, this study improves our knowledge of the relationship between the structural integrity and protective barrier functions of the airway epithelial layer. The *in situ* flow visualization and simulation achieved via tissue-wise particle tracking and computational modeling can collectively allow rapid analysis of the transport phenomena of the airway surface fluid. Direct measurements, coupled with computer simulation, of the electrical properties of both healthy and injured airway tissues can promote label-free, minimally invasive detection and quantification of airway tissue injury. Collectively, the outcome of the study offers innovative tissue monitoring tools and computational models that can facilitate both fundamental and translational studies associated with diagnosis and treatment of airway tissue injury.

## IV. METHODS

### A. Isolation of rat trachea

In this study, Sprague–Dawley rats (SAS SD Rats; Charles River Laboratories; weight: 200–250 g; number: 10) were used. The animals were handled in accordance with an animal protocol approved by the Institute for Animal Care and Use Committee (IACUC) at Stevens Institute of Technology. For the experiments, we used tracheas that were freshly isolated from the animals using our established methods.^37^ Briefly, the rats were euthanized by exposing them to 5% isoflurane for 30 min. After euthanasia, the tracheas were removed and gently washed using Dulbecco's modified Eagle medium: nutrient mixture F12 (DMEM/F12, CAT. No. 21041025, Gibco) to remove the blood from the tissue surface. The tracheas were left in the medium until they were used for the subsequent experiments.

### B. Generation of localized injury in the airway epithelium

To generate a localized physical injury in the epithelium, the isolated trachea was cut into two segments along the P–D axis of the trachea. Each segment of the trachea was trimmed to its final length of approximately 6 mm. The cilia of multiciliated epithelial cells were stained with fluorescein-labeled wheat germ agglutinin (FITC-WGA; Cat. No. FL-1021-5, Vector Laboratories; excitation/emission: 488/525 nm).^34,38^ Then, a small physical injury was created near the center of the tracheal lumen by gently compressing the tissue surface with the tip of a metal rod (diameter: 0.5 mm) that was mounted on a micropositioner. To improve visibility of the injured tissue region during subsequent live-tissue imaging, the tip of the metal rod was coated with 0.025% Evans blue solution (EB; CAT. No. 19555, Acros Organics; excitation/emission: 620/680 nm)^39^ before creating the epithelial injury. Both native and injured airway tissues were evaluated via scanning electron microscopy (SEM) and fluorescent microscopy.

### C. Scanning electron microscopy (SEM)

Airway tissue injury was confirmed by imaging the injured tissue region via scanning electron microscopy (SEM).^40,41^ Briefly, prior to imaging, the rat tracheas were fixed by immersing them in 2.5% glutaraldehyde (Cat. No.16520, Electron Microscopy Sciences) at 4 °C overnight. The fixed trachea tissues were rinsed thoroughly using phosphate-buffered saline (PBS) and dehydrated in a graded ethanol series. The tissues were further dehydrated by incubating them in 1,1,1,3,3,3-hexamethyldisilazane (Cat. No. 999-97-3, Acros Organics) overnight, followed by desiccation in a fume hood for one day. The tracheal tissues were then mounted on alumina stubs (Cat. No. 16111-9, Ted Pella) via carbon conductive tape (Cat. No. 16073, Ted Pella) and coated with gold (Cat. No. 12150EQ-AX, SPI Supplies) via sputtering for SEM imaging (Gemini SEM, Zeiss).

### D. Immunofluorescence staining

Immunostaining techniques were used to visualize the tight junctions, cilia, and Vangl-1 PCP protein of the airway tissue. Prior to immunostaining, trachea tissues were fixed in −20 °C methanol for 10 min, and then blocked in 10% fetal bovine serum (FBS) for 30 min at room temperature. To evaluate the tight junctions, the fixed tissues were incubated with anti-ZO1 tight junction protein primary antibody (Cat. No. ab190085, Abcam) overnight at 4 °C. The tissues were then incubated with secondary antibody (Cat. No. 705-025-147, Jackson ImmunoResearch Laboratories) for 1.5 h at room temperature. After secondary antibody, the tissues samples were incubated with DAPI solution (4′,6-diamidino-2-phenylindole; Cat. No. ab228549, Abcam) for 10 min to stain cell nuclei. Finally, the stained tissues were placed onto a glass slide with their luminal surface facing up. Antifade mounting medium (Cat. No. H-1700-10, Vector Laboratories) was applied to the tissue and a coverslip was gently placed on top of the tissue. After the mounting medium dried, the stained trachea tissues were imaged using a fluorescent microscope (LSM 880, Zeiss).

### E. Histological analysis

All tracheas, both native and injured tracheas, were fixed in 4% paraformaldehyde (PFA) solution at 4 °C overnight. Then, the fixed tracheas were dehydrated in a series of isopropyl alcohol solutions (IPA): 70%, 80%, 95%, and 100% and CitrisolvTM solution (Cat. No. 1061, Decon) prior to paraffin embedding. After paraffin embedding, the trachea tissues were cut into slices with thickness of 5–8 *μ*m, and then were deparaffinized using CitrisolvTM solution followed by rehydration in a series of IPA solutions: 100%, 95%, 90%, 80%, 70%, and 60%. After that, the tissue sections were stained for hematoxylin and eosin (H&E) using a H&E Staining Kit (Cat. No. ab245880, Abcam).

### F. Live imaging of cilia motion and mucociliary flow

For live imaging of cilia beating, we fluorescently labeled the ciliated cells with FITC-WGA.^34,38^ To do this, isolated rat trachea segments were placed in DMEM/F12 that contains 8 *μ*g/ml of FITC-WGA (Cat. No. FL-1021–5, Vector Laboratories; excitation/emission: 488/525 nm) for 1 h. Then, the luminal surface of the trachea was rinsed three times with DMEM/F12 to remove residual FITC-WGA. The trachea tissues were imaged immediately using an inverted microscope (Eclipse TE2000-E, Nikon) via 40× Plan Fluor objective lens (MRH08430, Nikon). Videos of beating cilia were acquired and recorded at 23 frames per second (fps) using a camera (pco.pixelfly usb, PCO). To visualize the flow generated by the cilia motion, fluorescent polystyrene microparticles (Cat. No. F13081, Invitrogen; excitation/emission: 540/560 nm; diameter: 1 *μ*m) mixed in DMEM/F12 culture medium (volume: 2 ml; microparticle concentration: 10^5^–10^6^ microparticles/ml) were gently applied onto the tracheal lumen. Videos and images of flow movement were acquired using a 20× Plan Fluor objective lens (MRH08230, Nikon).

### G. Imaging-based flow analysis

Flow speed was measured by analyzing the trajectories of the moving microparticles in the acquired videos. Analysis of the videos was achieved using the TrackMate function of ImageJ, which provided information about the trajectories, travel distance, and speed of microparticles at different time points. To determine the angular orientation of the flow in both native and injured tissues, the angle between the microparticle trajectory with respect to the P–D axis was measured via ImageJ. The histogram of angular direction of the microparticle movements were plotted using Oriana software. The index of orientation, which quantifies the degree of unidirectionality of microparticle movement, was calculated according to the following equation:

$$D=\frac{1}{N}\sum_{i=1}^{N}D_{i},$$ (1)

where *D_i_* represents the unit vector of each trajectory's displacement, *N* is the total number of trajectories detected by TrackMate, and *D* is served as the mean vector. The index of orientation is defined as |*D*| and in a range between 0 and 1, where 0 and 1 represent a random direction flow and unidirectional flow, respectively.^28^

### H. Computer simulation of the cilia-generated flow

For simulation of the cilia-generated laminar flow in injured airway surface, we used COMSOL Multiphysics^®^ 6.0. The flow was modeled as a thin three-dimensional (3D) surface flow with 6 mm in length, 4 mm in width, and 500 *μ*m in height. Water was used as the fluid for the simulation where the fluid properties at room temperature (viscosity: 1.001 mPa s; density: 1.00 g/cm^3^) were entered as variables. Relevant boundary conditions (BCs) were assigned at the surfaces of the flow model. In particular, “sliding wall” BC was assigned at the ciliated tissue surface while “no-slip” BC was assigned to the injured tissue. Extra fine mesh for the model was generated to improve the resolution of the simulation. The simulated ciliary flow was analyzed to determine flow speed, direction, and pressure via post-processing functions of the software.

### I. Bioimpedance measurement

Electrical properties of the rat trachea were measured using an LCR meter (E4980AL, Keysight). Specifically, we measured electrical resistance (R) and reactance (X) of the tissues at various alternating current (AC) frequencies, ranging between 250 Hz and 135 kHz. Bioimpedance (Z) of the tissues were calculated using the acquired R and X values via Z = R + jX. Four-probe measurement technique was employed to improve the accuracy of the measurements. Four tungsten electrodes (SE-T, Lucas Signatone; length: 30 cm; tip diameter: 5 *μ*m) were used in this study. To place the electrodes at precise locations during measurements, each electrode was mounted on a micropositioner (S-725SLM or S-725SRM, Lucas Signatone). Position of the electrode tip was monitored using our custom-built microscope, integrated with a 10× long working distance (LWD) objective lens (375-039, Mitutoyo), a tube lens (AC254-100-A-ML, Thorlabs), and a scientific CMOS camera (pco.panda 4.2, PCO).

### J. Analysis of bioimpedance parameters using Cole model

The Cole model used to analyze the measurement results provides the bioimpedance as

$$Z{\left(s\right)=R+\mathrm{jX}=R}_{\infty }+\frac{R_{0}-R_{\infty }}{1+{\left(j\omega \tau \right)}^{\alpha }},$$ (2)

where R_∞_ is the resistance at infinite AC frequency, R_0_ is the resistance at zero AC frequency (i.e., the direct current, DC), ω is the angular frequency, τ is the time constant of the dispersion, and α is an empirical exponent.^42,43^ Through nonlinear curve fitting, the data were fitted to a semi-circle curve with its center (x_0_, y_0_) and radius (r_0_). To minimize the curve fitting error, we used the least absolute deviation (LAD) method.^18^ The best fit was determined to be the one that generated the minimal value of the function:

$$f\left(x_{0},\;y_{0},\;r_{0}\right)=\sum_{i=1}^{n}\left|\sqrt{{\left(x_{i}-x_{0}\right)}^{2}+{\left(y_{i}-y_{0}\right)}^{2}}-r_{0}\right|,$$ (3)

where *n* is the number of data point. The parameters of the Cole model were then extracted from the curve.^18,19^ For example, the two points where the curve intersects with the horizontal axis (i.e., resistance axis) represent R_0_ and R_∞_, respectively. α is an empirical exponent that serves as a measure of the position of the circle center below the horizontal axis and can be calculated according to the relationship between α and θ:

$$\theta =\frac{\pi \alpha }{2},$$ (4)

where θ can be calculated from the geometrical relationship:

$$\text{sin }\theta =\frac{R_{0}-R_{\infty }}{2r_{0}}.$$ (5)

τ corresponds to the inverse of the angular frequency at the highest reactance:

$$\tau =\frac{1}{2\pi f_{c}},$$ (6)

where $f_{c}$ is the frequency at the highest reactance.^43,44^

### K. Bioimpedance simulation

The electric current physics module of COMSOL was used to simulate the bioimpedance of both native and injured airway tissues with varied AC frequencies between 10 Hz and 135 kHz. The tissue model (length: 6 mm, width: 4 mm, and height: 545 *μ*m) consisted of four distinct layers that represent the pulmonary surface liquid (thickness: 15 *μ*m), epithelium (thickness: 30 *μ*m), submucosa (thickness: 200 *μ*m), and cartilage (thickness: 300 *μ*m). In the tissue model with epithelium injury, a small cylindrical volume (diameter: 0.5 mm; height: 29 *μ*m) was incorporated into the epithelium that represents removed tissue from the mucosa. Four circles (diameter: 0.5 *μ*m), each of which represents the tissue regions that are in contact with the electrode, were created near the injury site. The electrical properties of the fluid and tissue layers were obtained from the literature.^45^ Fine mesh was created for the simulation, and the solution was obtained in frequency domain. Simulation results, such as impedance and volume impedance density, were calculated using existing methods.^46^ Briefly, sensitivity (S) was calculated to indicate how much the resistivity of a particular point influences the total impedance according to the following equation for a four-electrode system:

$$S=\frac{\vec{J_{CC}}\;\cdot \;\vec{J_{PU}}}{I_{CC}I_{PU}},$$ (7)

where $\vec{J_{CC}}$ and $\vec{J_{PU}}$ are the current density of the current carrying (CC) electrodes and potential pickup (PU) electrodes, respectively. $I_{CC}$ is the current in the CC electrodes and $I_{PU}$ is the reciprocal current in the PU electrodes.^47,48^ The volume impedance density (VID) can be obtained by multiplying the sensitivity with the complex resistivity (ρ) as shown in the following equation: VID=Sρ to find the contribution from each constituent of the model to the total impedance measured in this model.^46,49^ If the volume impedance density is integrated over all points in the measurand, then we get the measured impedance:

Z=∭VID dv. (8)

### L. Hypertonic saline inhalation injury

To produce a minor insult limiting to cilia or tight junctions, hypertonic saline (3% NaCl) was added to luminal surfaces of rat trachea tissues for 5 min at 37 °C. Then, the treated tissues were immediately used in imaging-based flow analysis or bioimpedance measurement as described in Sec. IV I or IV J, or fixed by 4% PFA solution overnight for H&E and immunofluorescence staining.

### M. Acidic fluid aspiration injury

The gastric aspiration model was constructed according to previous literature.^22^ Briefly, when rats were anesthetized but could react to a forceps toe inch, 1 cc syringe with 18 gauge needle filled with 0.5 ml air and 0.5 ml HCl solution (pH 1.5) were inserted into rat trachea (2–3 cartilage rings below the larynx) and then the instillation was instigated quickly. After 10 min post-injection, the rats were sacrificed, and the tracheas were isolated for subsequent H&E and immunofluorescence staining, imaging-based flow analysis, and bioimpedance measurement.

### N. Statistical analysis

In all experiments, each group was repeated at least three times and results were demonstrated as mean ± standard deviation, which is calculated from the result of each repetition. Statistically significant differences between groups were determined by two-group, two-tailed Student's t-test, or one-way analysis of variance (ANOVA) with Tukey's *post hoc* test when there were three or more groups. ^*^p < 0.05, ^**^p < 0.01, ^***^p < 0.001.

## SUPPLEMENTARY MATERIAL

See the supplementary material for additional data, including the microscopic images of the airway tissues (supplementary material Figs. 1 and 2), particle trajectories (supplementary material Fig. 3), computer simulation airway model and results (supplementary material Figs. 4–7), airway tissue image analysis (supplementary material Fig. 8), parameters used for the Cole–Cole bioimpedance model (supplementary material Table 1), and legends for supplementary videos.

## Data Availability

The data that support the findings of this study are available from the corresponding author upon reasonable request.
